# Robust and Efficient Frequency Estimator for Undersampled Waveforms Based on Frequency Offset Recognition

**DOI:** 10.1371/journal.pone.0163871

**Published:** 2016-10-04

**Authors:** Xiangdong Huang, Ruipeng Bai, Xukang Jin, Haipeng Fu

**Affiliations:** School of Electronic Information Engineering, Tianjin University, Tianjin, China; Cleveland Clinic Lerner Research Institute, UNITED STATES

## Abstract

This paper proposes an efficient frequency estimator based on Chinese Remainder Theorem for undersampled waveforms. Due to the emphasis on frequency offset recognition (i.e., frequency shift and compensation) of small-point DFT remainders, compared to estimators using large-point DFT remainders, it can achieve higher noise robustness in low signal-to-noise ratio (SNR) cases and higher accuracy in high SNR cases. Numerical results show that, by incorporating a remainder screening method and the Tsui spectrum corrector, the proposed estimator not only lowers the SNR threshold of detection, but also provides a higher accuracy than the large-point DFT estimator when the DFT size decreases to 1/90 of the latter case.

## Introduction

Frequency estimation of undersampled waveforms is widely encountered in radar detection [1] and distance estimation [2] etc. The mainstream approach to solve this problem is Chinese Remainder Theorem (CRT), since it is able to reconstruct a large number from its remainders modulo several moduli. For the CRT-based frequency estimators [3–6], the remainders are directly acquired from DFT peak bins of multiple channels, which means that the estimation accuracy heavily relies on the DFT frequency resolution. Hence, once the undersampling rates of individual channels are determined, to obtain a high estimation accuracy, the existing estimators [4–6] use large-point DFT to enhance the DFT frequency resolutions, which inevitably results in high computational complexity. Therefore, an efficient CRT-based frequency estimator with small-point DFT needs to be developed.

Unlike the CRT-based estimators with large-point DFT, an estimator with small-point DFT tends to incur weak noise robustness and low accuracy. On one hand, as is known, the noise energy is uniformly distributed over all the DFT bins. As the number of DFT bins decreases, the noise contamination on each DFT bin increases, thereby the noise robustness of the estimator is deteriorated. On the other hand, small-point DFT brings a coarse frequency resolution and thus degrades the accuracy.

To overcome these two difficulties, this paper proposes a scheme based on frequency offset recognition, which was ignored by the existed estimators [4–6]. The scheme is based on a discovery that, for any reconstruction channel, the underlying frequency offset information can be utilized to highlight the peak DFT bin.

Specifically, in low SNR cases, for any individual undersampled waveform, we use a performance index to recognize the value range of the frequency offset and then implement frequency-shift operation to highlight the peak DFT bin buried in noise. Moreover, we also employ a measure of remainder screening [6, 7] to further recognize those channels with reliable DFT spectra. In high SNR cases, we employ the Tsui spectrum corrector [8] to accurately estimate the frequency offset, thereby to overcome the drawback of the coarse DFT frequency resolution. Numerical results demonstrate that, with the above frequency-offset related measures, the proposed estimator can provide better noise robustness and higher accuracy than the existing estimators whose DFT lengths are about 90 times larger than ours.

This paper is organized as follows. Firstly, we present the estimation model and make the error analysis of the existed long-point DFT based estimators. Secondly, we propose a frequency estimator based on frequency shift and compensation, spectrum correction and remainder screening. Lastly, we present the simulation comparison which verifies the proposed estimator’s superiority over the existed estimators.

## Estimation Model and Error Analysis

### CRT-based Frequency Model

Consider a signal formulated as

$$\begin{array}{c} x\left(t\right)=a\;\text{exp}(j2\pi f_{0}t), \end{array}$$ (1)

where *f*_0_ is the high frequency to be determined and *a* is a non-zero complex amplitude.

To estimate *f*_0_, *L* A/D converters with undersampling rates *F*_1_ ∼ *F*_*L*_ (assume *F*_1_ < *F*_2_ < ⋯ < *F*_*L*_) are used to discrete *x*(*t*) in parallel. To meet the requirement of the closed-form CRT [9, 10], *F*_1_ ∼ *F*_*L*_ need to be integers such that

$$\begin{array}{c} F_{i}=M\Gamma_{i},\;\;\;i=1,\cdots ,L, \end{array}$$ (2)

where *M* is the greatest common divider (GCD) of *F*_1_ ∼ *F*_*L*_ and thus integers Γ_1_ ∼ Γ_*L*_ are pairwise coprime.

Then, the *i*-th (*i* = 1, …, *L*) discrete sequence with length *M* can be denoted as

$$\begin{array}{c} x_{i}\left(n\right)=a\;e^{j2\pi f_{0}/F_{i}n},\;n=0,\cdots ,M-1. \end{array}$$ (3)

Therefore, the relationship between *f*_0_ and the individual undersampling rates *F*_1_ ∼ *F*_*L*_ is formulated as

$$\begin{array}{c} \left\{\begin{array}{c} f_{0}=n_{1}F_{1}+\eta_{1}F_{1}\\ f_{0}=n_{2}F_{2}+\eta_{2}F_{2}\\ \;\;\vdots \\ f_{0}=n_{L}F_{L}+\eta_{L}F_{L} \end{array}\right., \end{array}$$ (4)

where *n*_*i*_ is the unknown folding integer and *η*_*i*_ is the normalized frequency (0 ≤ *η*_*i*_ < 1).

Clearly, Eq (4) constitutes a CRT model, in which *F*_1_ ∼ *F*_*L*_ are moduli and *η*_1_ *F*_1_ ∼ *η*_*L*_ *F*_*L*_ are remainders. In other words, once the normalized remainders *η*_1_ ∼ *η*_*L*_ are acquired, the closed-form CRT [9, 10] can determine the folding integers *n*_1_ ∼ *n*_*L*_ and thus yields the estimate of *f*_0_.

### Error Analysis of the Existed Estimators

To acquire *η*_*i*_, the estimators in [4–6] implement *F*_*i*_-point DFT (zeros are padded to the end of sequence) on *x*_*i*_(*n*), *i* = 1, …, *L*, and calculate *η*_*i*_ by searching out the peak DFT bin (assume it falls at *k* = *k*_*i*_), i.e.,

$$\begin{array}{c} \eta_{i}≐k_{i}/F_{i},\;k_{i}\in Z\;\text{and}\;k_{i}\in \left[0,M\Gamma_{i}-1\right]. \end{array}$$ (5)

In [4–6], *f*_0_ is assumed to be an integer (i.e., integer times of the frequency unit Δ*f* = 1 Hz), which ensures that *η*_*i*_ − *k*_*i*_/*F*_*i*_ = 0 if the peak bin is correctly searched out.

However, in practice, *f*_0_ also allows to be a real number (i.e., its fractional part is nonzero). In this case, *η*_*i*_ − *k*_*i*_/*F*_*i*_ ≠ 0, i.e., estimate error inevitably occurs. Furthermore, the DFT size *F*_*i*_ = *M*Γ_*i*_ is really a bit large, implying that high computational complexity is consumed.

## The Proposed Estimator

### Problems of A Small-point DFT Based Estimator

To reduce the complexity, we attempt to substitute *M*Γ_*i*_-point DFT with *M*-point DFT. Accordingly, the frequency unit Δ*f* increases from 1 Hz to *F_i_*/*M* = Γ*_i_* Hz, indicating that the *i*-th individual DFT spectrum gets much coarser. To ensure a sufficiently high accuracy, apart from the peak bin index *k* = *k*_*i*_, the fractional part of the remainder *η*_*i*_ *F*_*i*_ should also be taken into account. In this case, we have

$$\begin{array}{l} \eta_{i}=(k_{i}+\delta_{i})\Delta f/F_{i}=\left(k_{i}+\delta_{i}\right)/M,\\ \;k_{i}\in Z\text{ and }k_{i}\in \left[0,M-1\right],\;\delta_{i}\in \left[-0.5,0.5\right). \end{array}$$ (6)

The fractional number *δ*_*i*_ in Eq (6) refers to a normalized frequency offset, which was ignored by estimators in [4–6]. Clearly, estimation of *δ*_*i*_ can make up for the deficiency of coarse resolution of small-point DFT.

The other difficulty is the noise robustness. Specifically, for a same low SNR circumstance, compared to large-point DFT, the true peak bin of small-point DFT is more likely to be buried by heavy noise. As Eq (6) shows, compared to the estimate of *δ*_*i*_, the searching error of the integer peak index *k*_*i*_ will result in a larger estimation error of *η*_*i*_. In particular, the searching error of *k*_*i*_ might result in an incorrect estimate of the folding number *n*_*i*_, thus leading the CRT reconstruction into failure.

### Frequency Shift and Compensation

To improve the estimator’s robustness to noise, it is necessary to enhance the magnitude of peak DFT bin of *x*_*i*_(*n*) as possible.

Combining Eqs (3), (4) with (6), one can deduce *x*_*i*_(*n*) as

$$\begin{array}{c} x_{i}\left(n\right)=a\;e^{j(k_{i}+\delta_{i})2\pi n/M},\;n=0,...,M-1. \end{array}$$ (7)

Then, the normalized peak DFT bin *X*_*i*_(*k*_*i*_) can be derived as

$$\begin{array}{c} X_{i}\left(k_{i}\right)=a\cdot \frac{\text{sin}(\delta_{i}\pi )}{M\text{sin}(\delta_{i}\pi /M)}e^{j\delta_{i}\left(M-1\right)\pi /M}. \end{array}$$ (8)

**Fig 1:**
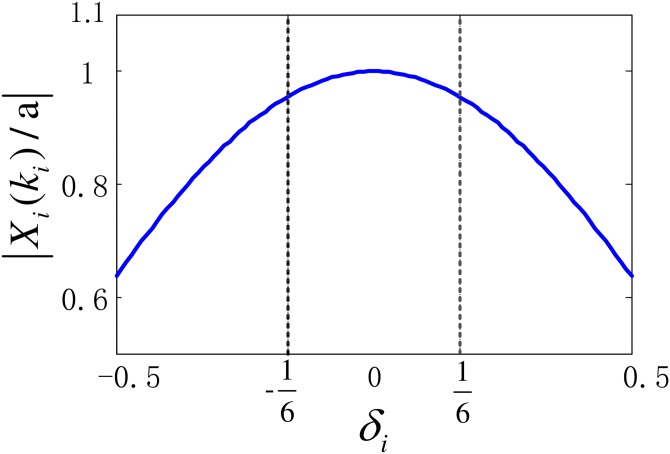
Normalized magnitude of peak bin versus *δ_i_*.

Eq (8) shows that, the magnitude of peak DFT bin is closely relevant to the frequency offset *δ*_*i*_. Fig 1 gives the curve of |*X*_*i*_(*k*_*i*_)/*a*| versus *δ*_*i*_.

From Fig 1, one can observe that as |*δ*_*i*_| increases, |*X*_*i*_(*k*_*i*_)/*a*| monotonously decreases, indicating that the DFT spectral leakage becomes increasingly more serious. As a result, for the low SNR case, it is likely that the peak DFT bin is buried.

To obtain a frequency offset approximating 0 as possilbe, we uniformly divide the value range $\left[-\frac{1}{2},\frac{1}{2}\right)$ of *δ*_*i*_ into three 1/3-length regions: $\left[\frac{1}{6},\frac{1}{2}\right),\left[-\frac{1}{6},\frac{1}{6}\right)$ and $\left[-\frac{1}{2},-\frac{1}{6}\right)$. For each region case, we attempt to construct another sequence with a stronger peak DFT bin through some frequency-shift operation. Therefore, we have the following discussions.

*1) Case of* $\delta_{i}\in \left[\frac{1}{6},\frac{1}{2}\right)$: We have $\left(\delta_{i}-\frac{1}{3}\right)\in \left[-\frac{1}{6},\frac{1}{6}\right)$, i.e., the middle region nearby 0. In terms of the property of Fourier transform, *x*_*i*_(*n*) needs to be applied with a left frequency-shift operation as

$$\begin{array}{c} x_{i}^{L}\left(n\right)=x_{i}\left(n\right)e^{-j\frac{1}{3}\cdot \frac{2\pi }{M}n},\;n=0,...,M-1 \end{array}$$ (9)

*2) Case of* $\delta_{i}\in \left[-\frac{1}{6},\frac{1}{6}\right)$: Since *δ*_*i*_ → 0 (meaning that the DFT spectral leakage is not serious), frequency-shift operation is not necessary, i.e., $x_{i}^{M}\left(n\right)=x_{i}\left(n\right)$.

*3) Case of* $\delta_{i}\in \left[-\frac{1}{2},-\frac{1}{6}\right)$: We have $\left(\delta_{i}+\frac{1}{3}\right)\in \left[-\frac{1}{6},\frac{1}{6}\right)$. Contrary to the case $\delta_{i}\in \left[\frac{1}{6},\frac{1}{2}\right)$, *x*_*i*_(*n*) should be applied with a right frequency-shift operation as

$$\begin{array}{c} x_{i}^{R}\left(n\right)=x_{i}\left(n\right)e^{j\frac{1}{3}\cdot \frac{2\pi }{M}n},\;n=0,...,M-1 \end{array}$$ (10)

Thus, given the normalized frequency ($\eta_{i}^{L},\eta_{i}^{M}$ or $\eta_{i}^{R}$) of the shifted sequence ($x_{i}^{L}\left(n\right),x_{i}^{M}\left(n\right)$ or $x_{i}^{R}\left(n\right)$), *η_i_* should be compensated by its frequency shift as

$$\begin{array}{c} \eta_{i}=\left\{\begin{array}{c} \eta_{i}^{L}+\frac{1}{3M},\;\delta_{i}\in \left[\frac{1}{6},\frac{1}{2}\right)\\ \eta_{i}^{M},\;\;\;\;\;\delta_{i}\in \left[-\frac{1}{6},\frac{1}{6}\right)\\ \eta_{i}^{R}-\frac{1}{3M},\;\delta_{i}\in \left[-\frac{1}{2},-\frac{1}{6}\right). \end{array}\right. \end{array}$$ (11)

However, the problem lies in judging which region *δ*_*i*_ falls in. Given the DFT spectra $X_{i}^{L}\left(k\right),X_{i}^{M}\left(k\right),X_{i}^{R}\left(k\right)$ of the above shifted sequences, we present a performance index defined by the ratio between the peak DFT bin and its adjacent sub-highest DFT bin, i.e.,

$$\begin{array}{c} \alpha^{c}=\frac{\left|X_{i}^{c}\left(k_{i}^{c}\right)\right|}{\max\left\{\left|X_{i}^{c}\left(k_{i}^{c}-1\right)\right|,\left|X_{i}^{c}\left(k_{i}^{c}+1\right)\right|\right\}}\;, \end{array}$$ (12)

where *c* ∈ {*L*, *M*, *R*}. Further, the region of *δ*_*i*_ can be recognized from the maximum of *α*^*L*^, *α*^*M*^, *α*^*R*^. Here, we present an example to illustrate this recognition criterion.

**Fig 2:**
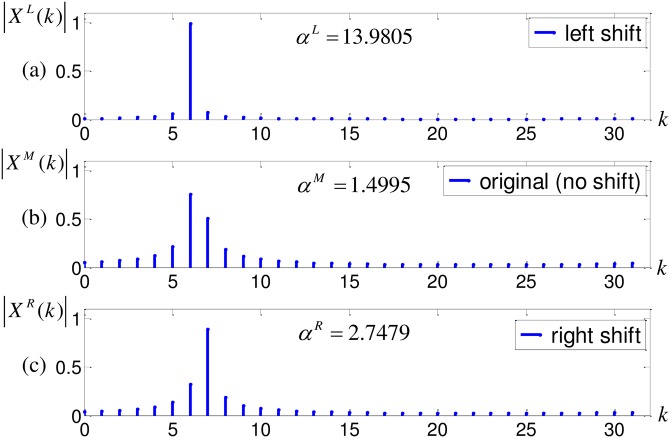
DFT spectra of different frequency shift cases.

***Example 1***: Consider a signal *x*(*n*) = exp(*j*2*π* ⋅ 6.4/*Mn*), *M* = 32, *n* = 0, …, *M* − 1. Ideally, the peak falls at *k* = 6 and *δ* = 0.4. Fig 2 gives the magnitude DFT specta |*X*^*L*^(*k*)|, |*X*^*M*^(*k*)|, |*X*^*R*^(*k*)| of their corresponding shifted sequences *x*^*L*^(*n*), *x*^*M*^(*n*), *x*^*R*^(*n*).

One can observe that, among 3 spectra, |*X*^*L*^(*k*)| in Fig 2(a) exhibits the smallest spectral leakage, i.e., its peak at *k* = 6 appears more conspicuous than others. Quantitatively, in terms of Eq (12), we have *α*^*L*^ = 13.9805, *α*^*M*^ = 1.4995, *α*^*R*^ = 2.7479. Thus, we can recognize that $\delta \in [\frac{1}{6},\frac{1}{2})$, which is in accord with the fact that *δ* = 0.4.

Further, in noisy circumstances, while the noise energy uniformly covers all DFT bins, the proposed frequency-shift operation still tends to highlight the peak DFT bin and thus yields a higher noise robustness.

### Spectrum Correction and Remainder Screening

The above recognition criterion can provide the reliable peak index *k*_*i*_ and the value range of the offset *δ*_*i*_. To improve the accuracy, *δ*_*i*_ needs to be further estimated. A lot of spectrum correctors such as Macleod correctors [11], Candan Correctors [12], Phase-difference correctors [13], Tsui correctors [8] are competent to estimate *δ*_*i*_ from the DFT result of *x*_*i*_(*n*). This paper suggests employing the Tsui spectrum corrector proposed in [8] (Its dataflow will be illustrated in the next section), since it currently possesses a high noise robustness and accuracy. Following this, all the CRT remainders ${\hat{r}}_{i}=\eta_{i}F_{i}=\left(k_{i}+\delta_{i}\right)F_{i}/M,\;i=1,...,L$, can be acquired.

Further, [5, 9] claimed that, the necessary condition of successful CRT reconstruction is

$$\begin{array}{c} |\Delta r_{i}\left|=\right|r_{i}-{\hat{r}}_{i}|<M/4,\;i=1,...,L, \end{array}$$ (13)

where *r*_*i*_ is the ideal errorless remainder. However, if the SNR is too low, not all *L* remainders satisfy this error bound condition.

Further, Ref. [6] provides a remainder screening method. Specifically, if there are ⌊(*L*−*K*)/2⌋ or fewer remainders exceeding the error bound Eq (13), this method can pick out these unrestricted remainders and use the remained remainders to achieve a successful CRT reconstruction. Accordingly, the CRT reconstruction range decreases from $f_{0}\in [0,M\prod_{i=1}^{L}\Gamma_{i})$ to $f_{0}\in [0,M\prod_{i=1}^{K}\Gamma_{i})$. This procedure of remainder screening will be listed in the next section.

### Procedure of the Proposed Estimator

The proposed frequency estimator based on frequency offset recognition is summarized as follows:

**Step 1** Implement 3 frequency-shift operations on the undersampled sequence *x*_*i*_(*n*), *i* = 1, ⋯, *L*, to generate the sequences $x_{i}^{L}\left(n\right),x_{i}^{M}\left(n\right),x_{i}^{R}\left(n\right)$ and their DFT results $X_{i}^{L}\left(k\right)$, $X_{i}^{M}\left(k\right)$, $X_{i}^{R}\left(k\right)$. Further, calculate their performance indices $\alpha_{i}^{L},\alpha_{i}^{M},\alpha_{i}^{R}$ in terms of Eq (12).
**Step 2** Use the frequency-offset based criterion to recognize the expected $X_{i}^{c}\left(k\right),c\in \left\{L,M,R\right\}$. Then, employ the Tsui spectrum corrector to obtain its normalized frequency estimate $\eta_{i}^{c}$. Further, use Eq (11) to obtain the compensated result *η*_*i*_. Thus, we can obtain all the remainders ${\hat{r}}_{i}=\eta_{i}F_{i},i=1,...,L$.
**Step 3** Substitute the moduli *F*_1_,…,*F*_*L*_, the remainders ${\hat{r}}_{1},...,{\hat{r}}_{L}$ and the specified integer *K* into the remainder-screening based CRT reconstruction procedure to obtain the final frequency estimate ${\hat{f}}_{0}$.

**Fig 3:**
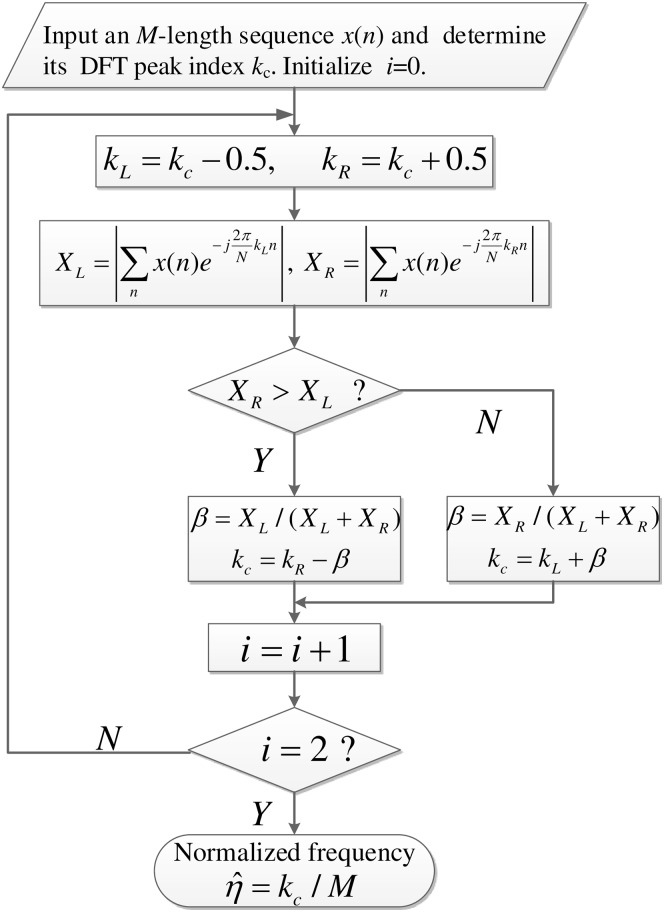
Dataflow of the Tsui spectrum corrector.

The procedure of the Tsui spectrum corrector addressed in Step 2 is illustrated in Fig 3.

The procedure of the remainder-screening based CRT reconstruction is listed in the following:

1. Employ ${\hat{r}}_{1}$ as the reference remainder and use the CRT remainders ${\hat{r}}_{1},...,{\hat{r}}_{L}$ to calculate the following *L* − 1 difference remainders ${\hat{q}}_{i,1}$:
  $$\begin{array}{c} {\hat{q}}_{i,1}=\left[\frac{{\hat{r}}_{i}-{\hat{r}}_{1}}{M}\right],\;2\leq i\leq L. \end{array}$$ (14)
2. Calculate the remainders of ${\hat{q}}_{i,1}{\bar{\Gamma }}_{i,1}$ modulo Γ_*i*_:
  $$\begin{array}{c} {\hat{\xi }}_{i,1}\equiv {\hat{q}}_{i,1}{\bar{\Gamma }}_{i,1}\;\text{mod}\;\Gamma_{i},\;2\leq i\leq L. \end{array}$$ (15)
  where ${\bar{\Gamma }}_{i,1}$ is the modular multiplicative inverse of Γ_1_ modulo Γ_*i*_ and can be calculated in advance.
3. Compute an intermediate integer *X*_1_ using the following formula
  $$\begin{array}{c} X_{1}\equiv \sum_{i=2}^{L}{\hat{\xi }}_{i,1}W_{i,1}\frac{\Gamma }{\Gamma_{1}\Gamma_{i}}\text{mod}\;\frac{\Gamma }{\Gamma_{1}}, \end{array}$$ (16)
  where Γ ≜ Γ_1_Γ_2_…Γ_*L*_, and *W*_*i*, 1_ is the modular multiplicative inverse of Γ/(Γ_1_ Γ_*i*_) modulo Γ_*i*_ that can be calculated in advance.
4. To determine the folding integer *n*_1_: Construct a set $Z=\{\prod_{\beta =1}^{L-\lfloor (L-K)/2\rfloor }\Gamma_{l_{\beta }}$, where Γ_*l*_1__, ⋯, Γ_*l*_*L* − ⌊(*L* − *K*)/2⌋__ is selected from all possible $\left(\begin{array}{c} \;L\\ \left\lfloor (L-K)/2\right\rfloor \end{array}\right)$ combinations enumerated among {Γ_1_, ⋯, Γ_*L*_}}. For every element *z* in **Z**, calculate
  $$\begin{array}{c} \hat{X}\left(z\right)\equiv X_{1}\;\text{mod}\;z. \end{array}$$ (17)
  If there is only one *z*_0_ in **Z** satisfying $0\leq \hat{X}\left(z_{0}\right)<\prod_{i=1}^{K}\Gamma_{i}$, let folding number ${\hat{n}}_{1}=\hat{X}\left(z_{0}\right)$.
5. To determine other folding integers *n*_*j*_, 2 ≤ *j* ≤ *L*: Exchange the reference remainder ${\hat{r}}_{1}$ with ${\hat{r}}_{j}$ and repeat 1)-4) to acquire the corresponding folding integers ${\hat{n}}_{j}$ respectively. It is possible that an individual *n*_*j*_ is null in an exchanged operation. Assume that altogether *P* (*P* ≤ *L*) times of operations are successful and their exchanged remainder indices are *v*_1_, …, *v_P_*. Thus, *P* frequency estimates ${\hat{f}}_{0}\left(\nu_{i}\right)={\hat{n}}_{\nu_{i}}F_{\nu_{i}}+{\hat{r}}_{\nu_{i}}$ for 1 ≤ *i* ≤ *P* can be obtained.
6. Remove those abnormal frequency estimates to form a reduced set $\Omega =\{{\hat{f}}_{0}\left(\mu_{1}\right),\;{\hat{f}}_{0}\left(\mu_{2}\right),\;\ldots ,\;{\hat{f}}_{0}\left(\mu_{Q}\right)\}$, in which any pair satisfy $|{\hat{f}}_{0}\left(\mu_{i}\right)-{\hat{f}}_{0}\left(\mu_{j}\right)|\leq M/2,1\leq i\neq j\leq P$. Further, take their average ${\hat{f}}_{0}=\frac{1}{Q}\sum_{i=1}^{Q}{\hat{f}}_{0}\left(\mu_{i}\right)$ as the final frequency estimate.

## Numerical Results

In this section, we compare the noise robustness and the accuracy among the large-point-DFT CRT estimator in [5] (LP-DFT estimator) and other 3 small-point-DFT CRT estimators: Tsui correctors with frequency shift (Tsui-FC estimator), Tsui correctors without frequency shift (Tsui estimator), and the proposed estimator. In this way, the improvement contribution of the frequency-shift operation, the spectrum correction and the remainder screening can be quantitatively investigated.

The parameters are as follows: The signal frequency *f*_0_ = 120000000.3Hz; The gcd *M* = 512; *L* = 6 undersampling rates:*F*_1_ = 73*M*, *F*_2_ = 79*M*, *F*_3_ = 89*M*, *F*_4_ = 97*M*, *F*_5_ = 101*M*, *F*_6_ = 103*M*; *K* = 4.

**Fig 4:**
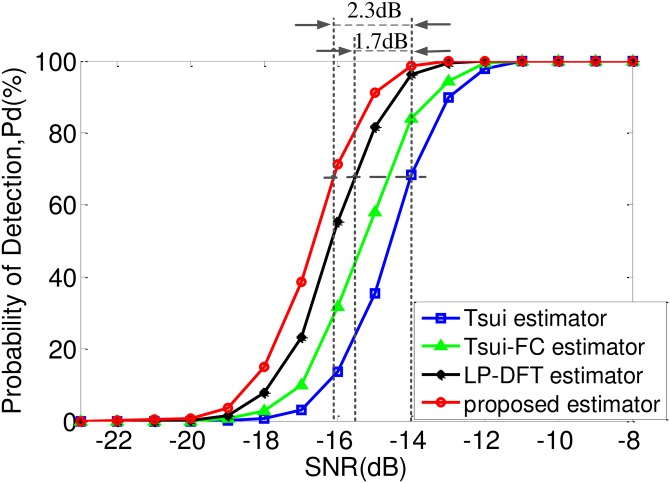
Detection probability curves of different CRT-based estimators.

***Example 2***: SNR varies in a low region [−23dB, −8dB]. For each SNR case and each estimator, 5000 Monte Carlo trials were conducted. We use the detection probability *P*_*d*_ to evaluate the noise robustness. Its criterion is as follows: For each trial, if ${\hat{f}}_{0}$ satisfies $|f_{0}-{\hat{f}}_{0}|<f_{0}/1000$, then this trial passes; otherwise, it fails. Fig 4 illustrates the *P*_*d*_ curves of these 4 estimators.

From Fig 4, the following conclusions can be drawn:

1. The proposed estimator (red circle curve) shows the best noise robustness, and the LP-DFT estimator (black diamond curve), the Tsui-FC estimator (green triangle curve), the Tsui estimator (blue rectangle curve) take the 2nd, 3rd, 4th place respectively, since their *P*_*d*_ curves successively appear from left to right.
2. The proposed estimator obtains 0.6dB SNR improvement than the LP-DFT estimator, since their *P*_*d*_ curves are about 2.3dB and 1.7dB left to that of the Tsui estimator, respectively. Specifically, as Fig 3 depicts, this improvement of our estimator arises from two contributions: 0.7dB stems from the frequency-shift operation and 1.6dB stems from the remainder screening [6].
3. Our proposed estimator concurrently possesses high noise robustness and high efficiency, since its DFT size *M* is about 90 times smaller than the average DFT size (*F*_1_ + … + *F*_6_)/6 of the LP-DFT estimator.

**Fig 5:**
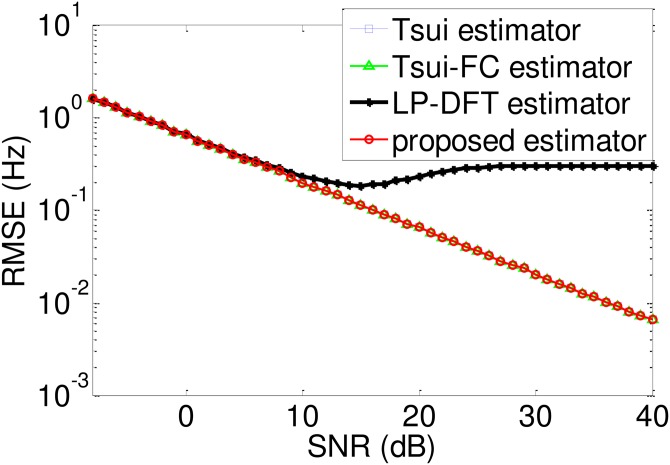
RMSE comparison among different CRT-based estimators.

***Example 3***: SNR varies in a higher region [−8dB, 40dB], in which all the 4 estimators can acquire 100%. detection probability. Fig 5 illustrates the root-mean-square error (RMSE) curves of these 4 estimators.

From Fig 5, the following conclusions can be drawn:

1. In the region where SNR ∈ [−8, 10] dB, all the small-point DFT estimators achieve the same the RMSE level of that of the large-point DFT estimator, which reflects that spectrum correction can alleviate the side effect of small-point DFT’s coarse resolution.
2. In the region where SNR > 10 dB, the RMSE curve of the proposed estimators is lower than that of the large-point DFT estimator, which exhibits a flat shape due to its fixed resolution. This reflects that, in high SNR cases, spectrum correction can lead to a higher accuracy.

## Conclusions

This paper proposes a small-point DFT based frequency estimator for undersampled waveforms, in which the recognition of the frequency offset *δ*_*i*_ plays a critical role in both high SNR cases and low SNR cases. Numerical results verifies the proposed estimators superiority in noise robustness, accuracy, and efficiency over the large-point DFT based estimator, which presents a vast potential for future applications.
